# Flakka-Induced Prolonged Psychosis

**DOI:** 10.1155/2016/3460849

**Published:** 2016-06-22

**Authors:** Craig Crespi

**Affiliations:** Palm Beach Consortium of Graduate Medical Education/University Hospital and Medical Center, 7201 North University Drive, Tamarac, FL 33321, USA

## Abstract

In South Florida, there has been a highly addictive new synthetic drug flooding the streets for people looking for a cheap high. Alpha-PVP, better known as Flakka, is an illegal substance that sells on the streets for as little as $5 a hit and delivers an instant high that can last from hours to days with lingering effects for weeks after it has been ingested. Although people use Flakka for its potential euphoric high, symptoms are known to easily escalate into frightening delusions, paranoid psychosis, extreme agitation, and a multitude of other altered mental states. According to the National Institute on Drug Abuse, Florida appears to be the nation's hot spot for reports of Flakka. In this case report, a 17-year-old female with no prior psychiatric diagnosis presents to the hospital under a 72-hour involuntary placement for altered mental status with agitation and psychotic behaviors. After multiple days of symptomatic treatment with benzodiazepines and antipsychotics, the patient became coherent enough to give a history of a “friend” putting Flakka in her food at school as a joke. Although she continues to have residual symptoms including psychomotor agitation and slowing of cognition, she was alert, oriented, and able to be discharged home with proper follow-up.

## 1. Introduction

Alpha-PVP (*alpha-pyrrolidinovalerophenone*), known on the streets as “Flakka,” is a new synthetic drug that has become an epidemic in South Florida. Flakka is the latest in a series of synthetic drugs that have become popular in the United States; included on this list are Ecstasy and Bath Salts. It is chemically similar to MDPV, also known as Bath Salts, which was blamed for a surge of bizarre cases of intoxication and agitation throughout the US a few years ago [1].

A part of the cathinone class, Flakka is a very addictive substance created in laboratories in order to produce euphoric symptoms in people trying to obtain a cheap, quick high. Cathinones have been found to stimulate the release of dopamine and inhibit the reuptake of epinephrine, norepinephrine, and serotonin in the central nervous system. Since cathinones are hydrophobic molecules, they can easily cross cell membranes and the blood brain barrier, allowing them to heavily interact with the monoamine transporters in the synaptic cleft between neurons [2].

Flakka is also known to provoke a condition called agitated delirium, when there is an excessive influx of sympathetic activation. This condition causes alterations in the mental status and can include bizarre behaviors, anxiety, agitation, violent outbursts, confusion, myoclonus, and rare cases of seizures. Clinical symptoms of agitated delirium involve tachycardia, hypertension, hyperthermia, diaphoresis, and mydriasis [3].

## 2. Summary of Case

Ms. C, a 17-year-old female with no past psychiatric diagnosis and who has never been seen by a mental health professional, presents to the psychiatric hospital under a Baker Act, a 72-hour involuntary placement, after being transferred from a local Emergency Department for altered mental status with agitation and psychotic behaviors, including auditory hallucinations.

The patient was originally brought to the hospital at the request of the patient's mother who noticed an acute onset of these bizarre behaviors. During the initial evaluation, the patient was drowsy and not coherent enough to give an accurate history of the events leading up to her current altered state. According to her mother, the patient was at home when she began yelling and screaming “go away!” while she was alone in her bedroom. The patient claimed that it was just a nightmare but the mother refutes that claim, saying that she was not sleeping and has no history of nightmares or sleep terrors.

The mother further denies any past history of any mood or psychotic disorders and believes that her daughter is just overwhelmed with stress due to multiple factors including school-related pressures and a long-distance relationship. The patient's laboratory values obtained at the Emergency Department were within normal limits besides her urine drug screen showing tricyclic antidepressants, which the mother explained that it could be due to a cream being used to treat the patient's migraines for many years.

## 3. Coarse of Hospital Stay

Since the patient has never experienced any symptoms of this nature in the past, she was admitted on day one for observation and symptomatic treatment without any routine psychotropic medications being started. On the following day, the patient needed full assistance from the staff with her activities of daily living and continued to act bizarre and illogical. Although unable to fully communicate due to her altered thought process, she mentioned that she might have been given Flakka by a “friend.” A noncontrast CT scan of the head was ordered to rule out any organic causes, which came back negative. Similar symptoms continued to be apparent on the third hospital day, where the patient remained bizarre, disorganized, and psychotic, repeating the phrase “Thank you, thank you Jesus.” She again mentioned that she might have taken Flakka but remains vague about the incident. Since the patient received intramuscular Olanzapine and Lorazepam multiple times since being admitted, it was deemed appropriate to start the patient on scheduled Olanzapine to target her symptoms. By the fifth hospital day, the patient was taking Olanzapine 10 mg twice a day routinely with Lorazepam every four hours as needed for agitation. Finally, on day six, the patient became coherent, alert and oriented to person, place, time, and situation, and capable of completing her activities of daily living. She remained somewhat constricted and at times required redirection and instructions to complete tasks. When asked about her symptoms for the past week, she described an incident that happened at school the day before being admitted to the hospital. She claims that a group of her “friends” were pressuring her to try Flakka with them. Although she refused, she believes that they put some on the food she was eating because she claimed it tasted funny and felt weird ever since. She also denies any recent major stressors or traumatic events that could have led to her behaviors. After one more day of observation, the patient did not display any more overt psychotic symptoms and was discharged home with the appropriate scheduled outpatient appointments.

## 4. Discussion

The street drug known as “Flakka” has been the latest plague of the synthetic substances causing havoc on the streets and in hospitals. South Florida is the epicenter of multiple Flakka episodes, with users displaying bizarre and psychotic behaviors [1]. In this case report, the patient's baseline mental status changed abruptly and drastically from only one use of Flakka. Even with the use of benzodiazepines and antipsychotics, the patient became alert and oriented but never returned back to her normal functioning.

Although the exact mechanism of action is unclear, why it has been causing this alteration in a person's functions, it is known that Flakka is designed to cause the brain to become flooded with dopamine. This influx in dopamine causes an intense feeling of euphoria but also leads a person to the possibility of agitated delirium and thus psychiatric hospitalizations [4]. Similar to most of the newer synthetic cathinones and other “legal high” compounds (e.g., synthetic cannabinoids K2 and Spice), Flakka is not detected by routine urine drug tests and can only be identified in select laboratories using gas chromatography and mass spectrometry [5].

The major challenges facing the clinicians managing a person with cathinone intoxication are control of agitation and other signs of sympathetic excess and acute decompensation can occur if immediate measures are not taken. Although most respond to aggressive treatment, the course is usually prolonged and many never return back to baseline. Until we can stop the import of the synthetic substance from international sources, the epidemic is likely to persist.
